# PRRT of neuroendocrine tumors: individualized dosimetry or fixed dose scheme?

**DOI:** 10.1186/s13550-020-00623-3

**Published:** 2020-04-15

**Authors:** Alexander R. Haug

**Affiliations:** 1 Division of Nuclear Medicine, Medical University of Vienna, Vienna, Austria; 2 Christian Doppler Laboratory for Applied Metabolimics, Vienna, Austria

**Keywords:** Neuroendocrine tumor, Dosimetry, 177Lu, DOTATATE, PRRT

## Abstract

Great efforts have been made in dosimetry for individualizing PRRT. However, many centers do not use dosimetry and its results hardly influence treatment. A reason for that is that reliable thresholds for organs-at-risk, kidneys and bone marrow, and treatment response are lacking. The nuclear medicine community must provide solid data from large trials delivering reliable thresholds, which then help to tailor PRRT according to organ doses (in order to reduce toxicity or increase treatment activity) or tumor doses (in order to increase activity to meet the response-threshold). Otherwise, development of radionuclide therapies will be done like big pharmaceutical companies do it currently: classical dose escalation studies and agreement on acceptable toxicity probabilities. Therapeutic radiopharmaceuticals will then be handled like other drugs, which on the other hand will increase availability of radionuclide therapies.

## Background

**Fig. 1:**
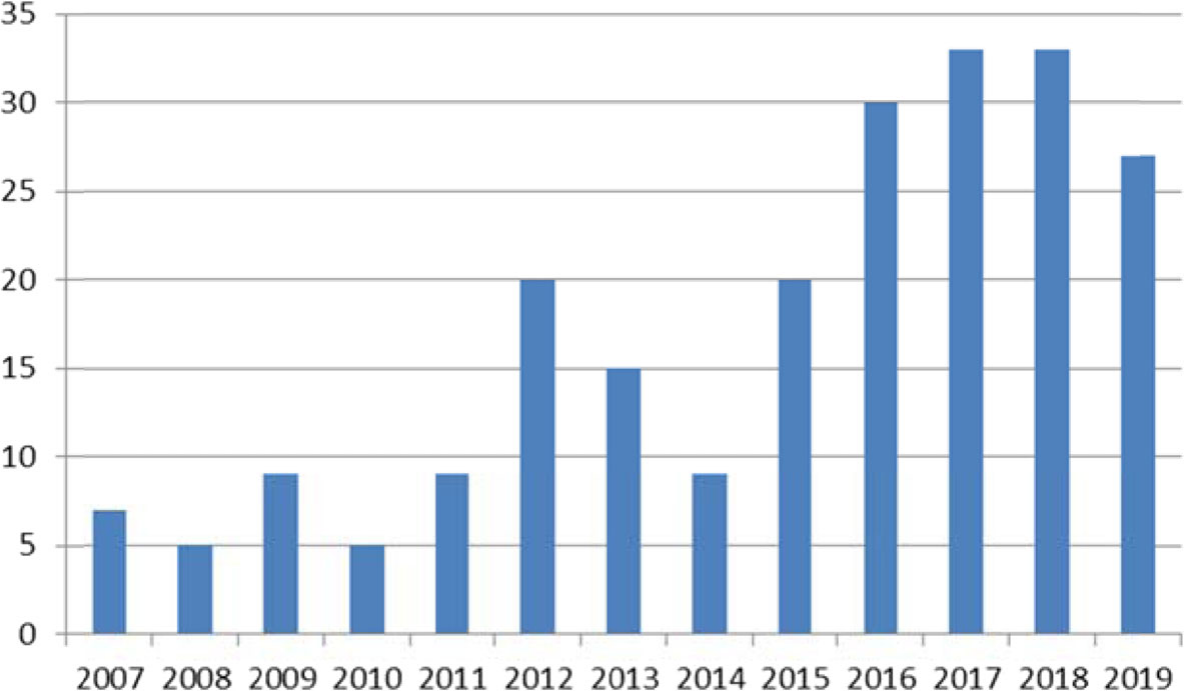
Number of publications per year using the search-terms “Dosimetry” and “177Lu*” on PubMed

In treatment of oncologic diseases, different treatment strategies have been implemented. Most therapies follow a fixed dose concept, administering a pre-defined fixed dose to a given patient. Typical examples for the fixed dose concept are not only targeted therapies such as sunitinib, everolimus, and erlotinib, but also radionuclide therapies such as most radioiodine therapies in thyroid cancer or PRRT with [^177^Lu]DOTATATE and [^177^Lu]PSMA. More patient-adapted treatments follow a body-weight- or body-surface-based concept, which calculates the dose to the patients´ weight or body-surface. Typical examples are some chemotherapeutics such as 5-FU and treatments with antibodies. A third concept modulates the treatment according to given clinical factors such as tumor stage and proven risk factors. Radiation therapy is partly an example, where the dose to a given tumor is intensified in case of risk factors such as high-risk prostate cancer or locally advanced head-and-neck cancer. Radioiodine therapy of thyroid cancer serves as another example, in which the administered activity is based on patient age, histological subtype, or T-, and N-stage. Nuclear Medicine has made great efforts in the recent past, particularly in order to further improve PRRT using dosimetry for a tailored treatment to an individual patient. These efforts have been translated into clinical routine, subsequently with the need for frequent costly scintigraphic measurements for measuring absorbed doses to critical organs. In order to justify these efforts, the impact of dosimetry on radionuclide therapy has to be evaluated carefully. Consequently, an increasing number of publications dealing with dosimetry of [^177^Lu] have been published in the last years (Fig. 1).

### Status quo

In contrast, a recent survey in European centers applying radionuclide therapies has shown, that dosimetry is hardly or never used in more than half of all centers [1]. Moreover, an individualized treatment activity was used only in 20% of all centers and in less than 10% activity was adjusted to organs at risk. These findings are most probably based on the following factors:

1. Dosimetry is time consuming, labor intensive, therefore expensive, and costs patient time.
2. In not gamma-emitting radionuclides such as [^90^Y] surrogate radionuclides such as [^111^Indium] are needed for dosimetry. There is a body of evidence that the obtained results are not well correlated to the therapeutic radionuclide, which hampers its useful clinical application.
3. In order to justify this effort an impact on treatment is warranted by defining (yet lacking) thresholds for

- Organs-at-risk in order to avoid toxicity
- Tumor response in order to improve efficacy

In radiation therapy, for example, thresholds for organs and tumor response are well established and both heavily influence treatment planning. In contrast, how is the situation in radionuclide therapy? In general, two organs at risk are considered in PRRT: the kidneys and bone marrow. Since PRRT using [^177^Lu] should generally be preferred over [^90^Yttrium] due to higher efficacy (no study has ever reported higher response rates for [^90^Y]DOTATATE) and less toxicity (especially regarding nephrotoxicity), this manuscript will solely focus on [^177^Lu]DOTATATE. Several studies have shown that higher-grade nephrotoxicity using [^177^Lu]DOTATATE is almost negligible with an annual loss of kidney function around 3.4% [2–4]. Nevertheless, a threshold of 23 Gy derived from radiation therapy is still used particularly from regulatory bodies. There is no evidence that this threshold is useful for PRRT. So far, no study has ever proven a clear correlation of kidney dose and nephrotoxicity. A recent study has shown that the biological equivalent dose to the kidneys does not correlate with the annual loss of glomerular filtration rate [5]. Further on, in this study some patients received up to 8 treatment cycles with 7.4 GBq [^177^Lu]DOTATATE without significant nephrotoxicity. The situation is quite the same with regard to hematotoxicity. The rate of higher-grade hematotoxicity is considered to be around 10% [2, 3]. In a study with 320 patients grade 3/4, hematotoxicity was correlated with clinical risk factor such as low leucocytes, impaired kidney function, age over 70 years, and extensive tumor mass [6]. Bone marrow dose showed a weak, but significant correlation with the change of leucocytes and platelets, but not with the change of hemoglobin. However, it was not possible to identify a certain threshold for the risk of developing hematotoxicity. Especially, the often used threshold of 2 Gy adapted from radioiodine therapy had no proven use. A reason for this finding might be the high variability of bone marrow dose depending on the method used. SPECT-based activity concentration fluctuated 100% (L5 median 24.2 kBq/ml, T11 median 47.8 kBq/ml) depending on the vertebral body examined [7]. Consequently, bone marrow doses varied depending on the used method: planar scintigraphy 0.19 Gy/7.4 GBq, SPECT of L4 0.36 Gy/7.4 GBq, SPECT of all visible vertebra 0,40 Gy/7.4 GBq, SPECT of all lumbar vertebra 0.39 Gy/7.4 GBq, and SPECT of all thoracic vertebra 0.46 Gy/7.4 GBq. In summary, neither for nephrotoxicity, nor for hematotoxicity thresholds have been reliably identified.

With regard to tumor response the situation is slightly better. For pancreatic neuroendocrine tumors a study showed a significant correlation between tumor dose and change in size [8]. A comprehensive methodology was used including repeated SPECT/CT and partial volume correction in 24 patients. Defining tumor shrinkage of 30% corresponding to RECIST partial remission as tumor response a threshold of 170 Gy was identified in tumors larger than 2.2 cm. In contrast, the same group found no correlation between tumor shrinkage and tumor dose (also not for change in tumor volume) in 25 patients with neuroendocrine tumors of the small intestine, despite using the same comprehensive methodology [9]. Notably, only 1/25 patients had partial remission as best response and median tumor shrinkage was 12%. The absorbed tumor dose was 128.6 Gy and comparable to that of pNET in the latter study. A correlation was, however, found between the administered activity and the tumor volume shrinkage (*p* = 0.01) and between the administered activity and RECIST 1.1. response (*p* = 0.01).

## Conclusion

Radionuclide therapy is currently on a crossroads between fixed treatment schemes and dosimetry-based individualized treatments. For the establishment of individualized PRRT, the currently quite variable dosimetry methods have to be harmonized, that means that the nuclear medicine community has to agree on an accepted and validated dosimetric methodology available for the majority of centers. Same is true for assessment of tumor response and follow-up of patients. In order to pave the road towards individualized treatment large prospective, randomized studies will be necessary identifying reliable and robust thresholds for toxicity and efficacy. These trials will have to prove that an individualized PRRT is superior to standardized PRRT with 4 × 7.4 GBq [^177^Lu]DOTATATE, as demonstrated in the NETTER trial [2].

If these developments will not happen, it is most likely development of radionuclide therapies will be done like big pharmaceutical companies do it currently: classical dose escalation studies and agreement on acceptable toxicity probabilities. The development of [^223^Radium] (Xofigo) by Bayer and [^177^Lu]DOTATATE (Lutathera) by AAA serve as examples of this approach. This approach will naturally neglect individual differences in organ and tumor doses. Therapeutic radiopharmaceuticals will then be handled like other drugs; on the other hand, requirements for users will be low which will increase accessibility of radionuclide therapy. Somewhat provocatively speaking, the implementation of this strategy will not significantly change the situation compared to the current status of radionuclide therapies, but the grade of evidence for it will markedly improve.

## Data Availability

Not applicable
